# Reversible cupping and persistent vessel narrowing after glaucoma surgery in childhood glaucoma: a quantitative fundus photograph study

**DOI:** 10.3389/fmed.2026.1794158

**Published:** 2026-04-20

**Authors:** Miao Zhang, Longyan Sun, Caixia Lin, Xiaowei Yu, Yan Shi, Zhigang Fan

**Affiliations:** Beijing Tongren Eye Center Research Ward, Beijing Tongren Hospital, Beijing Institute of Ophthalmology, Beijing Ophthalmology & Visual Sciences Key Laboratory Capital Medical University, Beijing, China

**Keywords:** childhood glaucoma, optic cup reversal, retinal vessel caliber, PPA regularity index, intraocular pressure

## Abstract

**Purpose:**

To investigate postoperative changes in peripapillary retinal vessel caliber and optic disc structure in childhood glaucoma.

**Design:**

Prospective, comparative cohort study with a 2-year follow-up.

**Methods:**

This study included 24 glaucomatous eyes (primary congenital or juvenile open-angle glaucoma) and 24 age-matched normal control eyes. Quantitative parameters, including vessel diameters (standardized to the vertical disc diameter), cup-to-disc ratios (CDR), rim widths, and β-zone parapapillary atrophy (PPA) morphology, were measured from standardized optic disc-centered fundus photographs using image analysis software (ImageJ).

**Results:**

Preoperatively, glaucomatous eyes demonstrated attenuated peripapillary vessel diameters, with the superior temporal vessels being significantly narrower than those in controls (*P* < 0.05). Postoperatively, despite successful IOP reduction (32.11 ± 8.36 to 14.30 ± 2.55 mmHg, *P* < 0.001), a paradoxical further narrowing of major peripapillary veins and arteries were observed which remained abnormally narrow compared to controls (*P* < 0.001). Although the mean vertical CDR change was not significant, 16.67% (4/24) of patients exhibited notable cup reversal. This reversal was strongly correlated with inferior rim widening (*R*^2^ = 0.84, *P* < 0.001). Preoperative disc hemorrhage and a higher β-zone PPA Regularity Index were independent predictors of cup reversal.

**Conclusion:**

Successful surgery unmasks a dichotomous remodeling response: a limited, biomechanical optic cup reversal in a subset of patients, linked to rim widening and an increase in PPA regularity, coexists with a persistent and paradoxical narrowing of the large retinal vessels. This indicates that vascular alterations may constitute a maladaptive or irreversible component of glaucomatous damage in children, distinct from the partially reversible connective tissue changes.

## Introduction

Glaucoma is a neurodegenerative disease characterized by damage to the optic nerve, commonly associated with elevated intraocular pressure (IOP). The optic nerve head (ONH)—the site where retinal ganglion cell axons exit the eye to form the optic nerve—is the primary locus of early and characteristic structural injury. In adult glaucoma, ONH remodeling arises from biomechanical–vascular coupling, wherein tissue deformation and impaired perfusion interact synergistically to promote axonal loss. In contrast, childhood glaucoma exerts unique biomechanical stress on the developing ONH, resulting in distinct and more diffuse optic cup enlargement. Unlike the localized cupping seen in adults—typically most pronounced in the superior-temporal or inferior-temporal regions—children often display generalized cupping that can partially reverse following IOP reduction. This reversibility reflects the greater tissue plasticity of the pediatric lamina cribrosa and peripapillary sclera. Optical coherence tomography (OCT) studies, such as that by Ely et al., have quantitatively demonstrated this phenomenon, highlighting the pediatric ONH as a dynamic structure capable of substantial biomechanical recovery.

However, the vascular adaptations of the pediatric ONH to elevated IOP remain poorly understood and may differ fundamentally from adult patterns, particularly in relation to the distinctive morphology of childhood cupping. Whether normalization of IOP induces a corresponding “vascular reversal” or whether vascular changes persist despite pressure reduction remains uncertain—a question of particular importance during ocular growth and visual development. Additional structural features, such as β-zone parapapillary atrophy (PPA), a potential marker of ONH resilience, and preoperative disc hemorrhage, an indicator of microvascular dysfunction, may also influence the ONH’s ability to undergo structural and vascular recovery. However, their relationships with postoperative remodeling in children have yet to be investigated. Accordingly, this study aims to investigate the coordinated structural and vascular responses of the pediatric ONH to both elevated and surgically reduced IOP by comparing eyes with childhood glaucoma to those of healthy controls. Our goal is to advance an integrated understanding of the biomechanical and vascular mechanisms that underpin ONH recovery in childhood glaucoma.

## Materials and methods

### Study design and participants

This prospective, comparative cohort study was conducted at the Beijing Tongren Hospital, Capital Medical University. The study was approved by the Ethics Committee of Beijing Tongren Eye Center and adhered to the tenets of the Declaration of Helsinki (1).

Medical records of all children aged 6 to 11 years diagnosed with primary congenital glaucoma (PCG) or juvenile open-angle glaucoma (JOAG) who underwent a successful 360° trabeculotomy at our institution between December 2022 and December 2023 were identified and included. Preoperative and 2-year postoperative examination data were collected, including best-corrected visual acuity (BCVA, converted to logMAR), IOP measured by Goldmann applanation tonometry, axial length assessed with the IOL Master 700 (Carl Zeiss Meditec AG, Jena, Germany), slit-lamp biomicroscopy, and stereo fundus photography (Topcon TRC-50EX, Tokyo, Japan). The diagnosis of PCG or JOAG was established based on standard clinical criteria: elevated IOP (> 21 mmHg), corneal abnormalities (e.g., edema, Haab’s striae in PCG), and characteristic optic disc cupping. Surgical success was defined as maintaining postoperative IOP ≤ 21 mmHg without the use of anti-glaucoma medications during a minimum follow-up period of two years.

The control cohort consisted of age-matched healthy children who presented for routine ocular examinations. To establish a longitudinal reference for normal development, these control subjects were also required to have completed a 2-year follow-up with available fundus photographs. They had no ocular disease other than mild refractive error, normal optic disc appearance, and IOP consistently ≤ 21 mmHg at all visits. The control group underwent identical ophthalmic examinations at two timepoints: baseline and after a matched follow-up period (about 2 years).

When both eyes met the inclusion criteria, only the left eye was included for analysis to ensure statistical independence. Key exclusion criteria for both groups included: (1) coexisting ocular diseases that could confound optic disc or retinal vessel assessment (e.g., optic nerve hypoplasia, retinopathy of prematurity, uveitis); (2) history of significant ocular trauma or prior intraocular surgery other than glaucoma surgeries; (3) media opacity precluding high-quality fundus photography; and (4) incomplete clinical or imaging data.

All 24 patients in the surgical cohort received preoperative topical antiglaucoma medications for IOP control prior to trabeculotomy. The median duration of preoperative medical therapy was 3.5 months (interquartile range: 2–6 months). The specific medication regimens were as follows: prostaglandin analog monotherapy (latanoprost 0.005% once daily, *n* = 8); β-blocker monotherapy (timolol 0.25% or 0.5% twice daily, *n* = 4); carbonic anhydrase inhibitor monotherapy (brinzolamide 1% twice daily or dorzolamide 2% three times daily, *n* = 2); dual combination therapy (prostaglandin analog + β-blocker, *n* = 6); and triple combination therapy (prostaglandin analog + β-blocker + carbonic anhydrase inhibitor, *n* = 4). Consistent with pediatric glaucoma safety guidelines, no patients received α2-agonists (brimonidine), which are contraindicated in children younger than 2 years due to risk of serious systemic adverse effects and require caution even in older children (2). For patients who had been on antiglaucoma medications prior to enrollment, a standardized washout period was observed before preoperative assessments: at least 4 weeks for prostaglandin analogs, and at least 2 weeks for β-blockers and carbonic anhydrase inhibitors. Postoperatively, consistent with our definition of surgical success (IOP ≤ 21 mmHg without medications), no antiglaucoma medications were used during the 2-year follow-up period.

### Fundus image analysis

**FIGURE 1:**
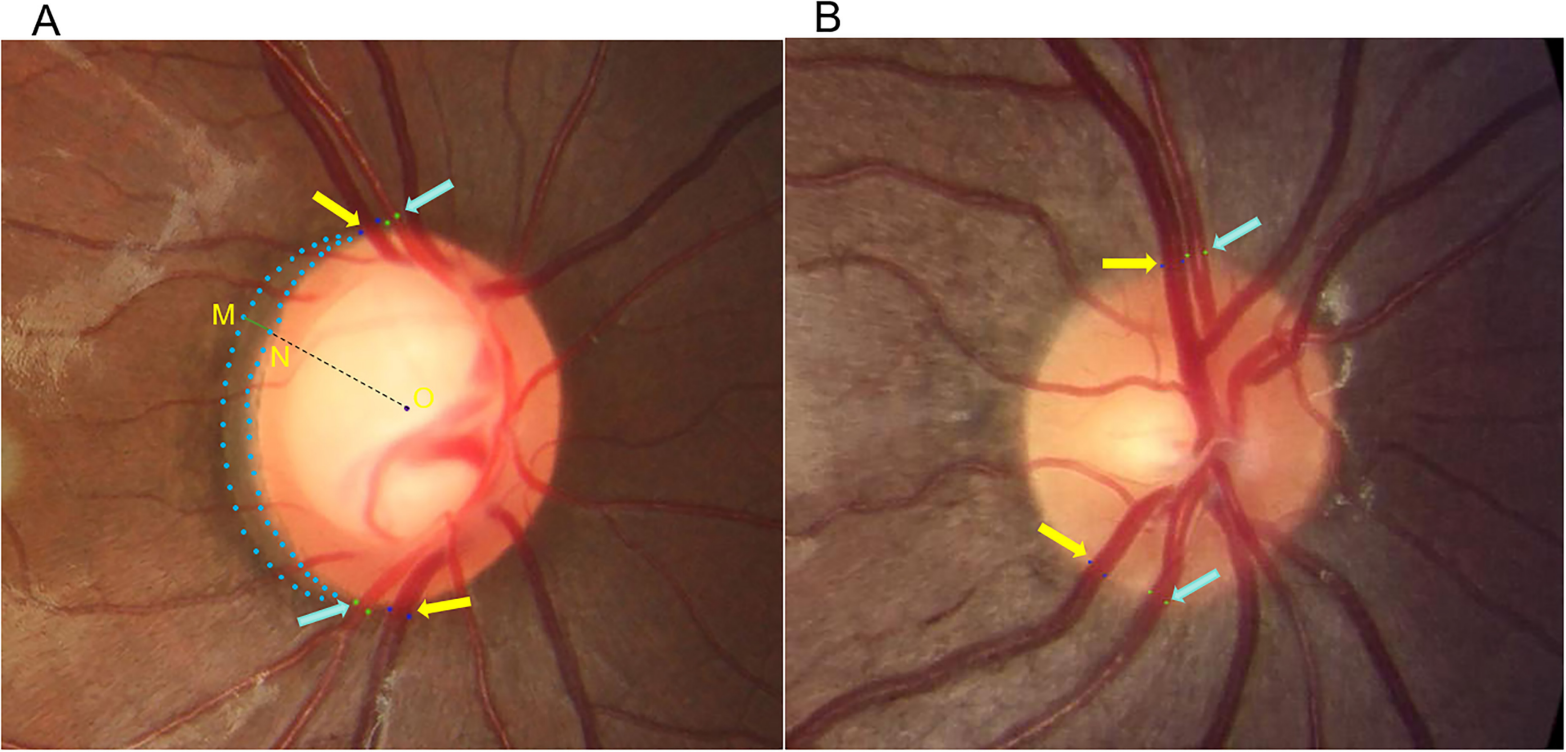
Schematic diagram of fundus image analysis and measurement protocol. **(A)** A representative preoperative fundus image from the glaucoma group. **(B)** A representative fundus image from the control group. Yellow arrows: superior and inferior temporal vein walls. Blue arrows: superior and inferior temporal artery walls. The blue dotted area outlines the β-zone parapapillary atrophy (PPA). Point O is the center of the optic disc. Points M and N define the maximum radial extent of the PPA and the optic disc margin, respectively. The distance between M and N is defined as the PPA Radial Extent. PPA Regularity Index was defined as the β-zone solidity measured by ImageJ.

Preoperative and postoperative stereo fundus photographs centered on the optic disc were analyzed. All measurements were performed using ImageJ software (National Institutes of Health, Bethesda, MD; version 1.53t) by two trained graders masked to the patient group and visit timepoint (3). The detailed measurement protocol is schematically illustrated in Figure 1. The presence of an optic disc hemorrhage was defined as a splinter-like or flame-shaped hemorrhage located on or immediately adjacent to the optic disc margin, as assessed on preoperative fundus photographs (4).

#### Measurement of optic disc and parapapillary structures

The margin of the optic cup was defined according to standard ophthalmoscopic criteria as the line delineated by the inward bend of the major retinal vessels as they cross from the cup floor to the neuroretinal rim. In ambiguous cases, the contour of the central pallor area served as a secondary reference (5). The vertical disc diameter (VDD) was measured as the maximum vertical distance across the disc margin. The vertical and horizontal cup-to-disc ratios (VCDR, HCDR) were measured. The neuroretinal rim width was measured at the superior, inferior, nasal, and temporal sectors.

The β-zone parapapillary atrophy (PPA) was defined as a chorioretinal atrophic area with visible sclera and choroidal vessels adjacent to the optic disc (6). The PPA radial extent was measured as the maximum distance from the disc margin to the atrophic border. The PPA area was outlined manually. To move beyond traditional size measurements and quantify the morphological regularity of the β-zone PPA, we defined a novel “PPA Regularity Index.” The PPA regularity index was calculated as the solidity of the PPA area (Area/Convex Area) using ImageJ software (National Institutes of Health), where a higher value indicates a more compact and regular shape (7). Solidity is a standard shape descriptor, calculated as the ratio of an object’s area to the area of its convex hull(8, 9). A value of 1 indicates a perfectly convex and regular shape, while values approaching 0 indicate increasing irregularity and concavity. This approach to quantifying tissue morphology is supported by studies highlighting the clinical relevance of connective tissue and laminar geometry in glaucoma pathophysiology(10, 11).

#### Measurement of retinal vessel diameters

The superior temporal artery and vein (STA, STV) and inferior temporal artery and vein (ITA, ITV) were identified. Their diameters were measured at the disc margin (for STAD, STVD, ITAD, ITVD) using the straight-line tool in ImageJ, calibrated to the scale of the image, a method validated in previous studies (6, 12). The arteriole-to-venule ratio (AVR) was calculated separately for the superior and inferior regions (Superior AVR = STAD/STVD; Inferior AVR = ITAD/ITVD) (13).

#### Normalization of measurements

To minimize magnification errors due to axial length differences and to allow for direct comparison, all linear measurements (vessel diameters, rim widths, PPA radial extent) were normalized to the vertical disc diameter (VDD) (12). Area measurements (PPA area) were normalized to the total optic disc area. This standardization approach is well-established in optic disc morphometry (7, 14). The normalized ratios are reported in all results.

### Assessment of measurement reproducibility

The intraobserver and interobserver reproducibility of all ImageJ-based measurements was rigorously assessed using the entire study cohort of 24 glaucoma patients (15). For intraobserver reproducibility, one observer remeasured all 24 preoperative fundus photographs on two separate occasions, with a 2-week interval. For interobserver reproducibility, a second independent observer measured the same set of 24 images. The reproducibility was evaluated using the intraclass correlation coefficient (ICC) with a two-way random-effects model for absolute agreement. ICC values were interpreted as follows: < 0.50, poor; 0.50–0.75, moderate; 0.75–0.90, good; and > 0.90, excellent (16).

### Statistical analysis

Statistical analyses were performed using Stata (Version 18.0; StataCorp, College Station, TX). For all quantitative measurements, the data from the first primary grader’s second measurement session were used for analysis to ensure consistency and minimize intra-observer variability. The normality of data distribution was assessed using the Shapiro-Wilk test. Continuous variables are presented as mean ± standard deviation or median [interquartile range (IR)]. Categorical variables are presented as numbers (percentages). Group comparisons were performed using independent samples t-test, Mann-Whitney U test, Chi-square test, paired t-test, or Wilcoxon signed-rank test, as appropriate. Univariate and multivariate line regression analyses were conducted to identify factors associated with the change in maximum cup width (defined as the value of the vertical cup diameters) and post-operative ITVD. Optic cup reversal was defined as a reduction in the maximum cup width by ≥ 0.1 from preoperative to postoperative measurement. A two-sided *P*-value of < 0.05 was considered statistically significant.

## Results

### Cohort characteristics and baseline comparison

The study cohort comprised 24 glaucomatous eyes (20 with primary congenital glaucoma, 4 with juvenile open-angle glaucoma) and 24 age-matched healthy control eyes. Preoperative demographic characteristics were comparable between the groups (Table 1). The mean age was 8.65 ± 2.32 years in patients versus 8.30 ± 0.33 years in controls (*P* = 0.60), with a similar sex distribution (*P* = 0.63). As expected, glaucomatous eyes exhibited significantly elevated intraocular pressure (IOP; 32.1 ± 8.4 vs. 13.6 ± 3.2 mmHg, *P* < 0.001), worse visual acuity (logMAR 0.68 (0.31–0.90) vs. 0.00 (0.00–0.00), *P* < 0.001), longer axial length (25.20 ± 0.46 vs. 23.42 ± 0.21 mm, *P* < 0.001), and larger corneal diameter (12.45 (12.00–14.00) vs. 12.09 (11.97–12.24) mm, *P* = 0.03). Optic discs showed profound glaucomatous damage, with larger vertical and horizontal cup-to-disc ratios (VCDR: 0.79 (0.64–0.91) vs. 0.27 (0.25–0.32); HCDR: 0.71 (0.64–0.88) vs. 0.26 (0.25–0.31); both *P* < 0.001). Preoperative disc hemorrhage was present in 4 (16.67%) glaucomatous eyes and absent in all controls. Quantitative vasculature analysis revealed attenuated normalized diameters of the superior temporal artery (STAD: 0.047 ± 0.01 vs. 0.055 ± 0.01, P = 0.03) and vein (STVD: 0.06 ± 0.01 vs. 0.07 ± 0.01, *P* = 0.004) in glaucoma eyes. No significant differences were found in inferior temporal vessel parameters or arteriole-to-venule ratios (all *P* > 0.05). Reliable measurement of β-zone parapapillary atrophy (PPA) was feasible in only six control eyes; therefore, formal intergroup comparison of PPA parameters was not performed.

**TABLE 1:** Baseline characteristics of the study cohort.

Characteristic	Glaucoma group (*n* = 24)	Control group (*n* = 24)	*P-*value
Age, years	8.65 ± 2.32	8.30 ± 0.33	0.60
Sex, male, n (%)	16 (66.7)	15 (60.0)	0.63
Mean IOP, mmHg	32.1 ± 8.4	13.6 ± 3.2	<0.001*
VA, logMAR	1.00 (0.52, 1.61)	0.00 (0.00, 0.00)	<0.001*
Axial length, mm	25.20 ± 0.46	23.42 ± 0.21	<0.001*
Corneal diameter, mm	12.45 (12.00, 14.00)	12.09 (11.97, 12.24)	0.03*
Preoperative VCDR	0.79 (0.64, 0.91)	0.27 (0.25, 0.32)	<0.001*
Preoperative HCDR	0.71 (0.64, 0.88)	0.26 (0.25, 0.31)	< 0.001*
Peripapillary retinal vessel diameter	–	–	**–**
STAD	0.047 ± 0.01	0.055 ± 0.01	0.03*
STVD	0.06 ± 0.01	0.07 ± 0.01	0.004*
Superior AVR	0.78 ± 0.12	0.76 ± 0.19	0.63
ITAD	0.049 ± 0.01	0.053 ± 0.01	0.15
ITVD	0.064 ± 0.01	0.070 ± 0.01	0.16
Inferior AVR	0.79 ± 0.14	0.80 ± 0.17	0.87

logMAR, logarithm of the minimum angle of resolution; IOP, intraocular pressure; VA, visual acuity; VCDR, vertical cup-to-disc ratio; HCDR, horizontal cup-to-disc ratio; STAD, superior temporal artery diameter; STVD, superior temporal vein diameter; ITAD, inferior temporal artery diameter; ITVD, inferior temporal vein diameter; PCG, primary congenital glaucoma; JOAG, juvenile open-angle glaucoma. All linear and area measurements are normalized to the optic disc dimensions (see Methods for details.

*Statistically significant (*P* < 0.05) was determined using independent samples *t*-test for normally distributed continuous data, Mann-Whitney U test for non-normal continuous data. Data are presented as mean ± standard deviation, median [interquartile range], or number (%), as appropriate. The control group has no follow-up duration as no surgery was performed. *Bold values indicate statistically significant differences (*P* < 0.05).

### Changes following glaucoma surgery

**FIGURE 2:**
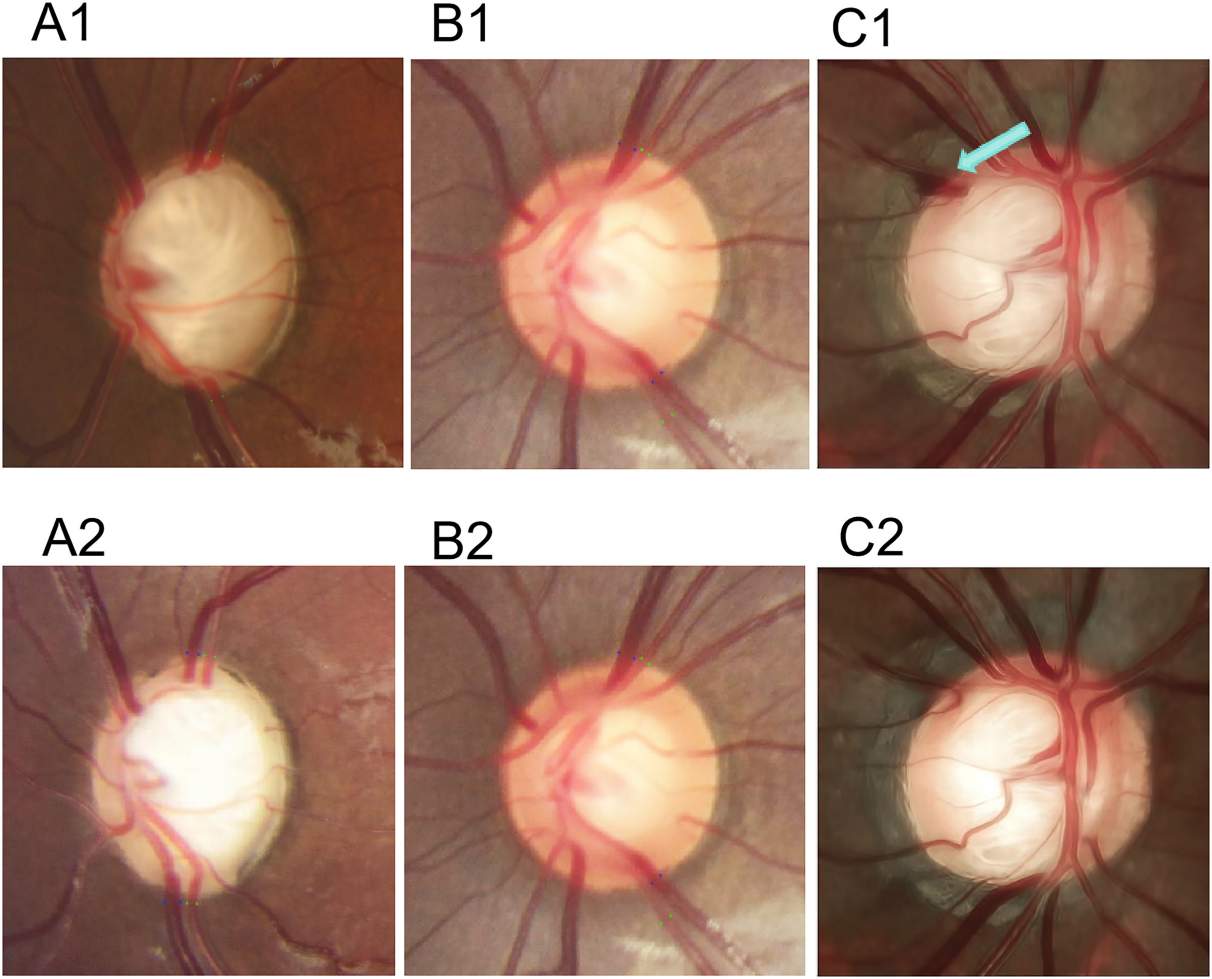
Representative cases with and without optic disc cup recession after surgery. Patient 1: **(A1)** Preoperative fundus photograph demonstrating a large optic cup. **(A2)** Postoperative fundus photograph showing noticeable recession of the optic cup. Patient 2: **(B1)** Preoperative fundus photograph. **(B2)** Postoperative fundus photograph showing no significant change in the optic cup size. Patient 3: **(C1)** Preoperative fundus photograph demonstrating a disc hemorrhage (indicated by the blue arrow). **(C2)** Postoperative fundus photograph showing that the disc hemorrhage has resolved.

**FIGURE 3:**
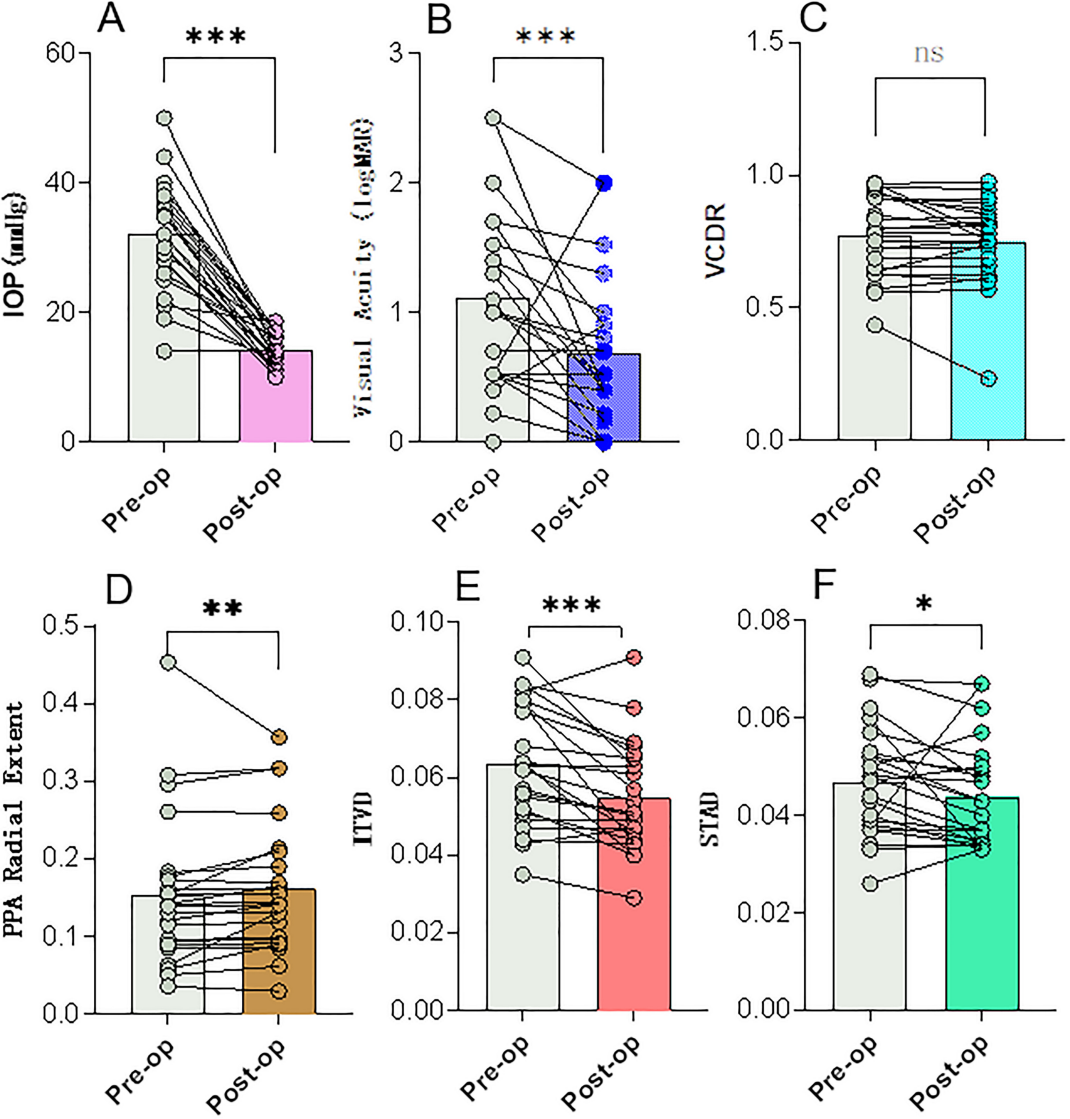
Changes in Key Ocular Parameters Following Glaucoma Surgery (*n* = 24). Box plots illustrate the preoperative (Pre-op) and postoperative (Post-op) distributions of **(A)** Intraocular pressure (IOP), **(B)** Visual acuity (logMAR), **(C)** Vertical cup-to-disc ratio (VCDR), **(D)** PPA radial extent, **(E)** Inferior temporal vein diameter (ITVD), **(F)** Superior temporal artery diameter (STAD). ***P < 0.001, **P < 0.001, *P < 0.05. ns, not significant.

After a median follow-up of 24.3 months, surgical intervention achieved a substantial reduction in IOP, from 32.1 ± 8.4 mmHg to 14.3 ± 2.6 mmHg (*P* < 0.001) (Table 2). Despite effective IOP control, a paradoxical narrowing of the retinal vasculature was observed postoperatively, alongside morphological progression of the β-zone PPA. The median VCDR (0.79 (0.64–0.91) to 0.77 (0.67–0.84), *P* = 0.38) and HCDR (0.71 (0.64–0.88) to 0.71 (0.64–0.81), *P* = 0.16) for the entire cohort showed mild changes. However, optic cup reversal (defined as VCDR reduction ≥ 0.1) occurred in 4 eyes (16.7%). Representative cases of optic cup reversal are shown in Figure 2. The β-zone PPA showed progression, with its radial extent increasing from 0.14 (0.09–0.18) to 0.14 (0.10–0.20) (*P* = 0.01) and its Regularity Index increasing from 0.34 (0.26–0.40) to 0.34 (0.28–0.45) (*P* = 0.04). Retinal vessels demonstrated significant constriction: the superior temporal artery diameter (STAD) decreased from 0.05 (0.038–0.052) to 0.04 (0.03–0.05) (*P* = 0.02), and the inferior temporal vein diameter (ITVD) decreased from 0.06 (0.05–0.08) to 0.05 (0.045–0.064) (*P* < 0.001) (Figure 3).

**TABLE 2:** Changes in ocular parameters following glaucoma surgery in the study cohort (*n* = 24).

Parameter	Preoperative	Postoperative	*P-*value
VA, logMAR	0.68 ± 0.57	1.11 ± 0.70	0.01*
IOP, mmHg	32.11 ± 8.36	14.30 ± 2.55	< 0.001*
Axial length, mm	25.20 ± 0.46	25.51 ± 0.53	0.12
VCDR	0.79 (0.64, 0.91)	0.77 (0.67, 0.84)	0.38
HCDR	0.71 (0.64, 0.88)	0.71 (0.64, 0.81)	0.16
Superior rim width	0.11 (0.08, 0.18)	0.11 (0.09, 0.18)	0.98
Inferior rim width	0.09 (0.02, 0.17)	0.11 (0.06, 0.16)	0.46
Nasal rim width	0.16 (0.12, 0.23)	0.18 (0.14, 0.24)	0.27
Temporal rim width	0.09 (0.02, 0.15)	0.10 (0.04, 0.14)	0.78
PPA radial extent	0.14 (0.09, 0.18)	0.14 (0.10, 0.20)	0.01*
PPA Area	0.20 (0.11, 0.30)	0.20 (0.15, 0.32)	0.15
PPA regularity index	0.34 (0.26, 0.40)	0.34 (0.28, 0.45)	0.04*
STAD	0.05 (0.038, 0.052)	0.04 (0.03,0.05)	0.02*
STVD	0.06 (0.05, 0.07)	0.055 (0.051,0.065)	0.08
Superior AVR	0.81 (0.72, 0.87)	0.75 (0.68, 0.86)	0.08
ITAD	0.048 (0.041, 0.056)	0.044 (0.039, 0.05)	0.06
ITVD	0.06 (0.05, 0.08)	0.05 (0.045, 0.064)	< 0.001*
Inferior AVR	0.82 (0.73, 0.90)	0.84 (0.71, 0.96)	0.28

logMAR, logarithm of the minimum angle of resolution; IOP, intraocular pressure; VA, visual acuity; VCDR, vertical cup-to-disc ratio; HCDR, horizontal cup-to-disc ratio; PPA, β-zone parapapillary atrophy; STAD, superior temporal artery diameter; STVD, superior temporal vein diameter; ITAD, inferior temporal artery diameter; ITVD, inferior temporal vein diameter. All linear and area measurements are normalized to the optic disc dimensions (see Methods for details). A higher PPA Regularity Index indicates a more compact and regular shape of the β-zone parapapillary atrophy.

*Statistically significant (*P* < 0.05). Data are presented as median (interquartile range) with Wilcoxon signed-rank test (non-normally distributed paired differences) or mean ± standard deviation with paired samples *t*-test (normally distributed paired differences), as appropriate. *Bold values indicate statistically significant differences (*P* < 0.05).

### Comparison with normal development and postoperative status

All ocular parameters in healthy controls (*n* = 24) remained stable over a matched median follow-up of 24.1 months (Table 3), confirming physiological stability during childhood. At the final visit, despite comparable IOP (14.3 ± 2.6 vs. 14.3 ± 2.5 mmHg, *P* = 0.20), surgical eyes exhibited persistent structural and vascular deficits (Table 4). Postoperative cup-to-disc ratios remained significantly larger (VCDR: 0.77 (0.67–0.84) vs. 0.30 (0.27–0.32); HCDR: 0.71 (0.64–0.81) vs. 0.28 (0.26–0.31); both *P* < 0.001). Crucially, all major peripapillary retinal vessels were persistently narrower than in healthy eyes: STAD (0.04 ± 0.01 vs. 0.06 ± 0.01, *P* < 0.001), STVD (0.06 ± 0.01 vs. 0.07 ± 0.02, *P* < 0.001), ITAD (0.046 ± 0.01 vs. 0.052 ± 0.01, *P* = 0.01), and ITVD (0.05 ± 0.01 vs. 0.07 ± 0.01, *P* < 0.001).

**TABLE 3:** Changes in ocular parameters over a 2-year follow-up in the normal control group cohort (*n* = 24).

Parameter	Baseline	2-year follow-up	*P-*value
VA, logMAR	0.00 (0.00, 0.00)	0.00 (0.00, 0.00)	1.00
IOP, mmHg	13.56 ± 3.18	14.25 ± 2.52	0.32
Axial length, mm	23.42 ± 0.21	23.69 ± 0.85	0.26
VCDR	0.27 (0.25, 0.32)	0.30 (0.27, 0.32)	0.46
HCDR	0.26 (0.25, 0.31)	0.28 (0.26, 0.31)	0.56
STAD	0.055 ± 0.01	0.056 ± 0.01	0.20
STVD	0.07 ± 0.01	0.07 ± 0.02	0.22
Superior AVR	0.76 ± 0.19	0.77 ± 0.23	0.46
ITAD	0.053 ± 0.01	0.052 ± 0.01	0.38
ITVD	0.070 ± 0.01	0.071 ± 0.01	0.85
Inferior AVR	0.80 ± 0.17	0.79 ± 0.21	0.25

logMAR, logarithm of the minimum angle of resolution; IOP, intraoular pressure; VA, visual acuity; VCDR, vertical cup-to-disc ratio; HCDR, horizontal cup-to-disc ratio; Retinal vessel diameters (STAD, superior temporal artery diameter; STVD, superior temporal vein diameter; ITAD, inferior temporal artery diameter; ITVD, inferior temporal vein diameter) are presented as the ratio of the absolute vessel diameter to the vertical optic disc diameter. AVR, arteriole-to-venule ratio. *Statistically significant (*P* < 0.05). Data are presented as median (interquartile range) with Wilcoxon signed-rank test (non-normally distributed paired differences) or mean ± standard deviation with paired samples *t*-test (normally distributed paired differences), as appropriate.

**TABLE 4:** Comparison of ocular parameters between the postoperative surgery group and the normal control group over a 2-year follow-up.

Parameters	Surgical group (postoperative) (*n* = 24)	Control group (*n* = 24)	*P*-value
VA, logMAR	0.61(0.31, 0.90)	0.00 (0.00, 0.00)	<0.001*
IOP, mmHg	14.30 ± 2.56	14.25 ± 2.52	0.20
VCDR	0.77 (0.67, 0.84)	0.30 (0.27, 0.32)	<0.001*
HCDR	0.71 (0.64, 0.81)	0.28 (0.26, 0.31)	<0.001*
STAD	0.04 ± 0.01	0.06 ± 0.01	<0.001*
STVD	0.06 ± 0.01	0.07 ± 0.02	<0.001*
Superior AVR	0.76 ± 0.11	0.77 ± 0.23	0.34
ITAD	0.046 ± 0.01	0.052 ± 0.01	0.01*
ITVD	0.05 ± 0.01	0.07 ± 0.01	<0.001*
Inferior AVR	0.84 (0.71, 0.96)	0.82 (0.65, 0.92)	0.48

logMAR, logarithm of the minimum angle of resolution; IOP, intraocular pressure; VA, visual acuity; VCDR, vertical cup-to-disc ratio; HCDR, horizontal cup-to-disc ratio; Retinal vessel diameters (STAD, superior temporal artery diameter; STVD, superior temporal vein diameter; ITAD, inferior temporal artery diameter; ITVD, inferior temporal vein diameter) are presented as the ratio of the absolute vessel diameter to the vertical optic disc diameter. AVR, arteriole-to-venule ratio.

*Statistically significant (*P* < 0.05). Data are presented as median (interquartile range) with Mann-Whitney U test or mean ± standard deviation with independent *t*-test, as appropriate. *Bold values indicate statistically significant differences (*P* < 0.05).

### Factors associated with postoperative remodeling

#### Predictors of optic cup change

Univariate linear regression analysis, with the change in vertical cup-to-disc ratio (ΔVCDR = postoperative - preoperative) as a continuous outcome variable, identified the presence of preoperative disc hemorrhage (β = −0.08, 95% CI: −0.13 to −0.02, *P* = 0.01) and a greater postoperative increase in the PPA Regularity Index (ΔPPA Regularity Index) (β = −1.07, 95% CI: −1.76 to −0.36, *P* = 0.004) as factors significantly associated with cup reduction (Table 5). In the multivariate linear regression model, both preoperative disc hemorrhage (β = −0.06, 95% CI: −0.11 to −0.002, *P* = 0.04) and ΔPPA Regularity Index (β = −0.83, 95% CI: −1.51 to −0.14, *P* = 0.02) remained independently significant. The negative regression coefficients indicate that the presence of preoperative hemorrhage and a larger increase in PPA regularity were each associated with a greater magnitude of cup reduction postoperatively (i.e., a more negative ΔVCDR value, indicating reversal).

**TABLE 5:** Univariate and multivariate line regression of changes in Max cup width (*n* = 24).

Variables	Univariate analysis			Multivariate analysis	
	Coefficient (95% CI)	*R*-squared	*P-*value	Coefficient (95% CI)	*P–*value
Age (years)	4.57 (−9.38, 18.51)	0.02	0.50	–	–
Sex	45.82 (−49.14, 140.78)	0.04	0.33	–	–
Pre-op axial length (mm)	−2.03 (−32.82, 28.20)	0.002	0.87	–	–
Pre-op mean IOP (mmHg)	−0.39 (−5.76, 4.98)	0.001	0.88		
Pre-op VCDR	−0.10 (−0.31, 0.12)	0.04	0.36		
Pre-op disc hemorrhage	−0.15 (−0.21, −0.10)	0.60	0.001*	−0.14 (−0.21, −0.07)	0.001*
Pre-op PPA regularity index	0.02 (−0.18, 0.21)	0.01	0.85	–	**–**
Post-op mean IOP (mmHg)	0.01(0.01, 0.02)	0.18	0.05		
Post-op ITVD	0.67 (10.03, 15.30)	0.03	0.14	–	**–**
ΔIOP (mmHg)	0.51 (−3.67, 4.70)	0.02	0.09	–	**–**
ΔITVD	−0.51(−3.60, 2.57)	0.06	0.73		
ΔPPA area	0.74 (−0.31, 1.78)	0.09	0.16		
ΔPPA regularity index	−1.07 (−1.76, −0.36)	0.31	0.004*	−0.10 (−0.15, −0.05)	0.04*
ΔPPA radial extent	0.55 (−0.55, 1.64)	0.06	0.31		

IOP, Intraocular Pressure; VCDR, vertical cup-to-disc ratio; PPA, β-zone parapapillary atrophy; ITVD, inferior temporal vein diameter. Pre-op, preoperative; Post-op, postoperative. All linear and area measurements are normalized to the optic disc dimensions (see Methods for details). A higher PPA Regularity Index indicates a more compact and regular shape of the β-zone parapapillary atrophy.

*Statistically significant (*P* < 0.05). Δ = Postoperative value − Preoperative value. *Bold values indicate statistically significant differences (*P* < 0.05).

#### Correlates of postoperative vascular changes

Analysis of the factors associated with postoperative retinal vein caliber (specifically ITVD) was performed (Supplementary Table 2). Univariate analysis showed that worse preoperative visual acuity, a larger preoperative ITAD, a larger preoperative ITVD, and a greater preoperative PPA radial extent were all significantly associated with the absolute value of postoperative ITVD. Notably, a larger preoperative ITVD was strongly correlated with a larger postoperative ITVD, suggesting a persistent state of venous narrowing. In the multivariate model, which excluded preoperative ITAD due to collinearity, only preoperative ITVD remained an independent correlate of the postoperative ITVD (coefficient: 0.55, *P* = 0.002).

**FIGURE 4:**
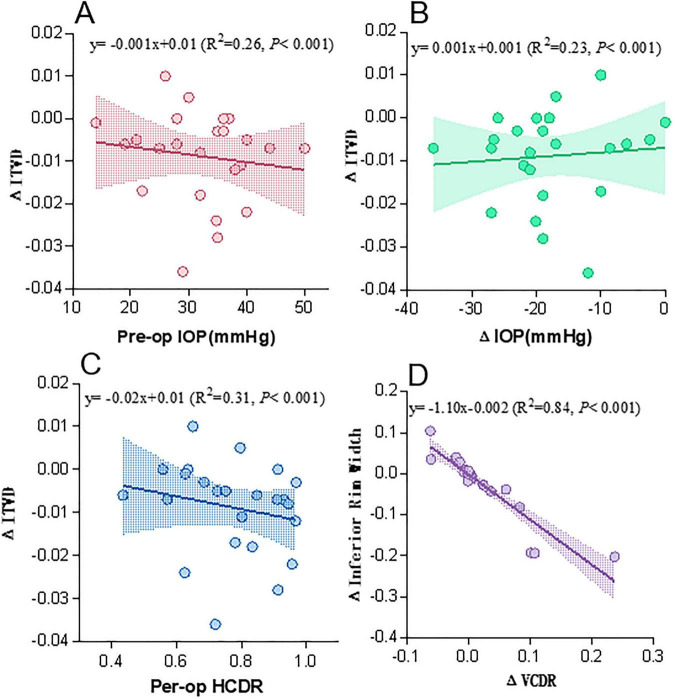
Correlations of postoperative changes (Δ, Postoperative -Preoperative) in optic disc structure and hemodynamics. **(A)** ΔITVD vs. preoperative intraocular pressure (IOP), **(B)** ΔITVD vs. ΔIOP, **(C)** ΔITVD vs. preoperative horizontal cup-to-disc ratio (HCDR), **(D)** ΔInferior rim width vs. Δvertical cup-to-disc ratio (VCDR). Pre-op, preoperative; Post-op, postoperative; ITVD, inferior temporal vein diameter; RW, rim width.

Furthermore, scatter plot analysis (Figure 4) elucidated relationships involving the change in ITVD (ΔITVD). A higher preoperative IOP was associated with more postoperative venous constriction (i.e., a less negative ΔITVD) (*R*^2^ = 0.26, *P* < 0.001; Figure 4A). Conversely, a greater reduction in IOP (ΔIOP) was associated with more pronounced venous constriction (*R*^2^ = 0.23, *P* < 0.001; Figure 4B). Eyes with more severe preoperative structural damage (larger preoperative HCDR) also associated with more postoperative venous constriction (R^2^ = 0.31, *P* < 0.001; Figure 4C).

### Measurement reproducibility

The image analysis protocol demonstrated excellent reliability. Both intraobserver and interobserver intraclass correlation coefficients (ICC) exceeded 0.90 for the vast majority of optic disc and retinal vessel parameters (Supplementary Table 1).

### Measurement reliability

The reproducibility of our image analysis protocol was excellent. Both intraobserver and interobserver intraclass correlation coefficients (ICC) exceeded 0.90 for the majority of optic disc and retinal vessel parameters, confirming the robustness of our measurements (Supplementary Table 1).

## Discussion

This study delineates the complex remodeling of the optic nerve head following surgical IOP control in childhood glaucoma. Our analysis reveals a multifaceted response where structural, vascular, and connective tissue components demonstrate distinct plasticity. The key findings are: a limited incidence of significant optic cup reversal (16.7%); a paradoxical, persistent constriction of the retinal vasculature postoperatively; and morphological changes in the β-zone PPA, with both preoperative disc hemorrhage and increased PPA regularity predicting greater cup reduction. Crucially, the stability of all parameters in age-matched healthy controls confirms these observations as pathological remodeling, not physiological development.

The limited incidence of cup reversal and its topographic specificity underscore the complex determinants of structural recovery. Using a criterion of ≥ 0.1 reduction in vertical cup-to-disc ratio (VCDR) to define significant cup reversal, we observed this phenomenon in 16.7% of patients (4/24). This proportion differs from the 50% reported by Ely et al. (17), potentially due to variations in study population characteristics. Notably, three of the four patients showing reversal had primary congenital glaucoma (PCG), with a mean preoperative VCDR of 0.90, suggesting that PCG may possess greater structural plasticity and that severe structural damage does not necessarily preclude reversal. Interestingly, neither preoperative IOP nor the magnitude of IOP reduction showed significant correlation with cup reversal in our cohort. This indicates complex determinants of structural recovery in childhood glaucoma. The relationship between IOP reduction and cup reversal may not be linear but rather threshold-dependent, where achieving a critical IOP level initiates structural changes, while the ultimate extent of reversal depends on multiple factors including surgical timing, duration of glaucomatous damage, and individual connective tissue properties (18–22). Irreversible fibrotic remodeling of the lamina cribrosa and peri-papillary tissues may limit structural recovery despite adequate IOP control (23). The limited sample size may also have affected our ability to detect significant correlations.

Topographic specificity is observed in neuroretinal rim recovery, with a predilection for the inferior sector. Our finding of a strong correlation between cup reversal and inferior rim widening suggests a topographically specific recovery pattern. The inferior neuroretinal rim is biomechanically more vulnerable to glaucomatous damage due to the larger, more compliant pores in the corresponding lamina cribrosa(24, 25); this inherent susceptibility may paradoxically facilitate greater potential for elastic recoil and mechanical restitution upon IOP reduction. In the context of childhood glaucoma, where axial elongation imposes significant mechanical stress, the typically broad inferior rim may possess a greater structural reserve for re-expansion compared to other sectors. Concurrently, vascular factors may contribute, as the microvasculature in the inferior peripapillary region, often severely affected in glaucoma (26, 27), might experience pronounced reperfusion postoperatively, thereby supporting structural recovery. Thus, the pronounced recovery of the inferior rim likely represents a confluence of its unique biomechanical properties, structural reserve, and associated vascular microenvironment.

The most striking finding is the postoperative retinal vasoconstriction. Our study reveals a central paradox in the vascular response to surgical intraocular pressure (IOP) reduction in childhood glaucoma: the persistence and even progression of retinal vessel narrowing despite successful IOP normalization. Interpreting this finding requires reconciling two key analytical results that, at first glance, may appear contradictory but are in fact complementary: (1) Correlation analyses (Figure 4) demonstrated that eyes with more advanced preoperative damage (larger cup-to-disc ratio, higher IOP) exhibited a smaller magnitude of postoperative venous constriction (ΔITVD); and (2) Multivariate regression (Supplementary Table 2) identified preoperative ITVD as the sole independent predictor of the absolute postoperative ITVD value, indicating that the ultimate vascular caliber is predominantly predetermined by its preoperative state.

Normal ocular development cannot explain this phenomenon. Our longitudinal control data confirm the stability of retinal vessel diameters over a 2-year period in healthy children, aligning with reports of a vascular caliber plateau in childhood. In a longitudinal study of Chinese children aged 2–6 years, Liu et al. (28) reported that vascular diameter changes occurred before the age of 4 years, followed by a period of stability with no significant changes observed between the ages of 4 and 6 years. This establishes that the observed changes in the glaucoma cohort are pathological.

These two key results address complementary aspects of vascular behavior. Figure 4 elucidates “vascular reactivity”—the dynamic, functional response to IOP change. The attenuated constriction in eyes with advanced disease implies diminished autoregulatory reserve or a proximity to maximal structural narrowing, limiting further functional contraction. In contrast, Supplementary Table 2 reveals profound “structural stability.” It demonstrates that irrespective of the functional response magnitude, the ultimate postoperative caliber is inextricably linked to the preoperative baseline. A vessel that is severely narrowed preoperatively will remain so postoperatively.

Studies by Jonas et al. (6, 29) and Mitchell et al. (13) established retinal vessel narrowing as a characteristic of chronic glaucomatous damage. Superimposed upon this fixed, narrowed state may be a secondary, functional component of autoregulation. According to established principles of retinal hemodynamics, vessels dilate to maintain perfusion under elevated IOP. Zhang et al. (30) demonstrated that acute IOP elevation produces differential venous responses: significant venous dilation with mild IOP increases (< 6 mmHg), moderate dilation with intermediate increases (6–15 mmHg), and no significant change with substantial increases (> 15 mmHg). Complementing this, Flammer’s theory of retinal vascular autoregulation (31) suggests that vessels constrict following acute IOP reduction to maintain stable blood flow.

The absence of postoperative vessel dilation in our pediatric cohort stands in contrast to some studies in adult glaucoma, where pharmacological IOP reduction has been associated with improved vessel density. Lestak et al. demonstrated that in newly diagnosed POAG patients, 3 months of treatment with carteolol significantly increased peripapillary vessel density in multiple segments, whereas latanoprost improved vessel density only in a single segment (32). This observation suggests that certain medications, particularly those with intrinsic sympathomimetic activity like carteolol, may have direct vasoactive effects independent of IOP lowering, potentially modulating vascular tone or endothelial function. In contrast, our study evaluated the effect of surgical IOP reduction alone, without any pharmacological vasoactive intervention.

This discrepancy likely reflects fundamental differences in vascular pathophysiology between pediatric and adult glaucoma. In children, chronic exposure to elevated IOP during a critical period of ocular growth may induce more permanent structural alterations in the vessel wall—a concept we term “vascular memory.” Long-standing IOP elevation can lead to remodeling of vascular smooth muscle, thickening of the basement membrane, and altered extracellular matrix composition, rendering the vessels incapable of dilation even after the removal of mechanical stress. In adults, the vasculature may retain greater functional plasticity, allowing for measurable improvements in perfusion following IOP reduction (23). These age-dependent and mechanism-dependent differences in vascular response warrant further investigation using comparative studies across pediatric and adult populations.

To integrate these observations, we propose a two-component pathophysiological model characterized by a “structural ceiling” that limits functional adaptation. The first and dominant component is a fixed, structural remodeling of the vessel wall. Chronic exposure to elevated IOP likely induces alterations in extracellular matrix composition and vascular smooth muscle function, establishing a new, narrowed structural set point. This irreversible remodeling explains the strong baseline dependency (Supplementary Table 2) and the permanent failure to normalize vessel diameter. The second component is a superimposed, transient functional autoregulatory response. Preoperatively, vessels may be in a state of compensatory dilation to maintain perfusion. The acute postoperative IOP reduction triggers physiological constriction to avoid hyperperfusion. However, the extent of this functional constriction is intrinsically limited by the pre-existing structural narrowing (23, 33, 34). Thus, eyes with less severe preoperative structural compromise (higher baseline ITVD) retain a greater capacity for functional constriction (explaining the correlations in Figure 4), yet their final caliber remains closely linked to their better baseline (as per Supplementary Table 2).

This model carries important clinical and pathophysiological implications. First, it highlights the prognostic value of preoperative vascular assessment. Baseline vessel caliber serves as a powerful predictor of the postoperative vascular architecture and may indicate the eye’s residual hemodynamic adaptive reserve. Second, it underscores a fundamental divergence in plasticity between tissue components of the pediatric optic nerve head. While the scleral and laminar connective tissues can demonstrate significant biomechanical recovery (e.g., cup reversal), the large retinal vessels appear to acquire a more permanent pathological signature. This relative “vascular memory” positions peripapillary vessel diameter as a potential stable biomarker of chronic glaucomatous stress, distinct from metrics of acute biomechanical change.

This persistent vascular narrowing raises an important clinical question: despite successful IOP control, do these children remain at risk for ischemia-related optic nerve damage? While our study did not directly assess perfusion, the fixed nature of the vascular narrowing suggests that these eyes may have limited capacity for autoregulatory compensation under future physiological stressors (e.g., nocturnal hypotension, systemic dehydration). Therefore, long-term monitoring of these children should extend beyond IOP measurement to include periodic assessment of peripapillary vessel caliber and, where available, OCT angiography to evaluate microvascular integrity. Whether these vascular changes translate into clinically significant visual outcomes warrants investigation in future longitudinal studies.

A key consideration raised is the potential confounding influence of myopia on our observations of retinal vascular changes. Indeed, our glaucoma cohort had significantly longer axial length (25.20 ± 0.46 mm) than controls (23.42 ± 0.21 mm), consistent with the well-established association between childhood glaucoma and axial elongation due to IOP elevation during ocular development.

Multiple studies have demonstrated that myopic eyes exhibit reduced peripapillary and macular vessel density compared to non-myopic eyes. Zeng et al., using swept-source OCT angiography, reported that high myopes exhibit lower vessel density and lower flow area, particularly in the superficial layer and the nerve fiber layer (35). Kayabaşi et al. further demonstrated that axial length is significantly associated with vascular density parameters across multiple capillary plexuses (36). Xu et al. recently showed that the retinal microvasculature of myopic eyes with axial length ≥ 26 mm exhibits greater susceptibility to physiological stressors, suggesting reduced vascular reserve (37).

The mechanisms underlying myopia-related vascular changes likely include mechanical stretching during axial elongation, structural remodeling of the sclera and choroid, and altered autoregulatory capacity. In eyes with both myopia and glaucoma, these vascular changes may be compounded. Therefore, the persistent vascular narrowing observed in our postoperative cohort likely represents a composite effect of glaucomatous microvascular damage and myopia-related vascular remodeling. These two processes may interact synergistically, with the myopic vascular phenotype predisposing the eye to more severe glaucomatous vascular injury. This observation underscores the importance of considering axial length as a confounding factor in future studies.

Within this framework, the transformed prognostic significance of preoperative disc hemorrhage becomes insightful. The identification of preoperative disc hemorrhage as an independent predictor favorable for cup reversal contrasts markedly with its established role as a risk factor for progression in adult glaucoma. While Airaksinen et al. (4) and Siegner and Netland (38) established disc hemorrhage as a risk factor for progression in adults, our results suggest it may indicate reparative potential in children. This differential significance likely reflects distinct pathophysiological bases. In childhood glaucoma, disc hemorrhage may represent acute microvascular stress response to IOP elevation rather than chronic progressive damage. This acute insult appears to trigger robust tissue repair and remodeling processes. Supporting this interpretation, all preoperative hemorrhages had resolved by postoperative examination, and these patients demonstrated superior structural recovery, indicating preserved reparative capacity in pediatric optic nerve tissue (39).

Our analysis revealed that postoperative **regularization** of β-zone PPA morphology correlates strongly with greater cup reversal. Both univariate and multivariate analyses confirmed that an increase in the PPA regularity index (solidity) is an independent positive predictor of cup reversal. The negative coefficient confirms a positive relationship since diff-VCDR values are negative when reversal occurs. This finding extends the work of Huh et al. (7), who associated irregular PPA morphology with glaucoma progression. Our results demonstrate that postoperative regularization predicts structural recovery, suggesting that PPA morphology reflects the biomechanical state of peri-papillary tissues. An increase in regularity may indicate several beneficial processes: tissue reperfusion following IOP normalization, redistribution of mechanical stress away from focal weak points, and resolution of reversible components like edema or inflammation. Thus, PPA regularization likely represents positive biomechanical remodeling of the supporting tissues surrounding the optic nerve head, working in concert with cup reversal to indicate effective recovery from glaucomatous injury (8, 40).

## Limitations

Several limitations should be acknowledged. First, the relatively modest sample size (*n* = 24) limits the statistical power of our multivariate regression analyses. Therefore, the results of these models should be interpreted with caution, as the number of variables examined is relatively high relative to the sample size. Future studies with larger cohorts are needed to validate these findings. Second, the single 2-year follow-up precludes assessment of long-term remodeling trajectories. Third, the use of fundus photography instead of optical coherence tomography angiography (OCTA) is a methodological limitation, as OCTA is the gold standard for assessing retinal microvasculature. However, obtaining high-quality OCTA images is challenging in some children due to poor cooperation. Future studies are expected to employ OCTA for more comprehensive investigations to address this limitation. Fourth, significant axial length differences between groups introduce potential myopia-related confounding, which may not be fully resolved by normalization to disc diameter. Future studies with larger, axial length-matched cohorts and advanced imaging are needed.

## Conclusion

This study demonstrates that childhood glaucoma surgery triggers complex structural and vascular remodeling beyond simple cup reversal. The limited prevalence of significant reversal, the consistent vascular constriction, and the dynamic PPA changes collectively indicate multifaceted adaptive mechanisms following IOP normalization. Preoperative disc hemorrhage may indicate reparative potential in children, contrary to its significance in adults. Postoperative retinal vessel constriction may reflect a combination of physiological autoregulation upon a background of fixed structural narrowing, rather than ongoing pathological progression. Most notably, an increase in the PPA regularity index serves as a positive predictor of structural recovery, reflecting beneficial biomechanical remodeling. These findings provide new insights into the mechanisms of optic nerve head recovery in childhood glaucoma.

## Data Availability

The raw data supporting the conclusions of this article will be made available by the authors, without undue reservation.
