# Supplementary material for: Reversible cupping and persistent vessel narrowing after glaucoma surgery in childhood glaucoma: a quantitative fundus photograph study
Source: Front Med (Lausanne). 2026 Apr 20;13:1794158. doi: 10.3389/fmed.2026.1794158 (PMC13136014; doi:10.3389/fmed.2026.1794158)
Supplement: Supplementary file 1 [file Table_1.docx]

**Table S1. Intraobserver and Interobserver Reproducibility of Optic Disc and Retinal Vessel Parameters Measured from Fundus Photographs (n=24).**

| **Parameter** | **Intraobserver ICC (95% CI)** | **Interobserver ICC (95% CI)** |
| --- | --- | --- |
| STAD / VDD | 0.96 (0.91–0.99) | 0.95 (0.94–0.99) |
| STVD / VDD | 0.93 (0.88–0.95) | 0.92 (0.87–0.94) |
| ITAD / VDD | 0.90 (0.86–0.95) | 0.88 (0.82–0.92) |
| ITVD / VDD | 0.92 (0.87–0.96) | 0.91 (0.86–0.96) |
| Superior AVR | 0.94 (0.90–0.97) | 0.92 (0.87–0.96) |
| Inferior AVR | 0.93 (0.89–0.96) | 0.91 (0.86–0.95) |
| VCDR | 0.96 (0.93–0.98) | 0.95 (0.92–0.98) |
| HCDR | 0.94 (0.90–0.99) | 0.93 (0.89–0.95) |
| Superior Rim Width / VDD | 0.89 (0.84–0.92) | 0.87 (0.84–0.90) |
| Inferior Rim Width / VDD | 0.95 (0.92–0.97) | 0.93 (0.89–0.96) |
| Nasal Rim Width / VDD | 0.95 (0.91–0.97) | 0.94 (0.90–0.97) |
| Temporal Rim Width / VDD | 0.84 (0.80–0.88) | 0.83 (0.81–0.85) |
| PPA Radial Extent / VDD | 0.92 (0.88–0.95) | 0.91 (0.86–0.97) |
| PPA Area / Disc Area | 0.92 (0.88–0.96) | 0.91 (0.86–0.95) |
| PPA Regularity Index | 0.97 (0.95–0.99) | 0.96 (0.93–0.98) |

 ICC, intraclass correlation coefficient; CI, confidence interval; STAD, superior temporal artery diameter; STVD, superior temporal vein diameter; ITAD, inferior temporal artery diameter; ITVD, inferior temporal vein diameter; AVR, arteriole-to-venule ratio; VCDR, vertical cup-to-disc ratio; HCDR, horizontal cup-to-disc ratio; VDD, vertical disc diameter; PPA, β-zone parapapillary atrophy.

Data are presented as ICC (95% CI). All measurements were normalized to the vertical disc diameter (VDD) or disc area. ICC values were interpreted as follows: <0.50, poor; 0.50–0.75, moderate; 0.75–0.90, good; >0.90, excellent.

**Table S2. Univariate and multivariate line regression of** **Post-op ITVD (n=24)**

| **Variables** | **Univariate analysis** | | | **Multivariate analysis** | |
| --- | --- | --- | --- | --- | --- |
|  | **Coefficient (95% CI)** | ***R-squared*** | ***P* value** | **Coefficient (95% CI)** | ***P* value** |
| Age (years) | 0.01 (-0.01, 0.01) | 0.02 | 0.46 | - | - |
| Sex | -0.01 (-0.02, 0.01) | 0.08 | 0.19 | - | - |
| Pre-op Axial length (mm) | -0.01 (-0.01, 0.01) | 0.001 | 0.98 | - | - |
| Pre-op Mean VA, logMAR | -0.01 (-0.02, -0.01) | 0.19 | **0.04*** | -0.01 (-0.01, 0.01) | 0.38 |
| Pre-op Mean IOP (mmHg) | -0.01 (-0.01, 0.01) | 0.003 | 0.77 | - | - |
| Pre-op VCDR | -0.02 (-0.06, 0.02) | 0.04 | 0.36 | - | - |
| Pre-op ITAD | 0.74 (0.38, 1.10) | 0.45 | **<0.001*** | a | a |
| Pre-op ITVD | 0.67 (0.40, 0.84) | 0.55 | **<0.001*** | 0.55 (0.23, 0.86) | **0.002*** |
| Pre-op PPA Area | -0.03 (-0.06, 0.01) | 0.12 | 0.12 | - | - |
| Pre-op PPA Regularity Index | -0.01 (-0.05, 0.03) | 0.01 | 0.60 | - | - |
| Pre-op PPA Radial Extent | -0.06 (-0.12, -0.01) | 0.17 | **0.047*** | -0.03 (-0.08, 0.01) | 0.15 |
| Post-op Mean IOP (mmHg) | 0.01 (-0.01, 0.01) | 0.02 | 0.47 | - | - |
| ΔIOP (mmHg) | 0.01 (-0.01, 0.01) | 0.01 | 0.61 | - | - |
| ΔPPA Area | -0.08 (-0.29, 0.12) | 0.03 | 0.40 | - | - |
| ΔPPA Regularity Index | 0.13 (-0.02, 0.27) | 0.13 | 0.09 | - | - |
| ΔPPA Radial Extent | 0.13 (-0.07, 0.33) | 0.08 | 0.19 | - | - |
| logMAR, logarithm of the minimum angle of resolution; IOP, intraocular pressure; VA, visual acuity; VCDR, vertical cup-to-disc ratio; PPA, β-zone parapapillary atrophy; ITAD, inferior temporal artery diameter; ITVD, inferior temporal vein diameter. Pre-op, preoperative; Post-op, postoperative. All linear and area measurements are normalized to the optic disc dimensions (see Methods for details). A higher PPA Regularity Index indicates a more compact and regular shape of the β-zone parapapillary atrophy.  *Statistically significant (*P* < 0.05). a.Because of collinearity with Pre-op ITVD, Pre-op ITAD was not included in the multivariate regression analysis.  Δ = Postoperative value - Preoperative value. | | | | | |

**Figure S1. Comparison of Parameters among groups.**

Data are presented as mean ± standard deviation. Statistical significance was determined by Kruskal-Wallisest lowed by Dunnett's test (vs. control group at baseline) and paired t-test (preoperative vs. postoperative). ***P < 0.001, **P < 0.01, *P < 0.05. ns, not significant.
