# Supplementary figures and images for: Reversible cupping and persistent vessel narrowing after glaucoma surgery in childhood glaucoma: a quantitative fundus photograph study
Source: Front Med (Lausanne). 2026 Apr 20;13:1794158. doi: 10.3389/fmed.2026.1794158 (PMC13136014; doi:10.3389/fmed.2026.1794158)

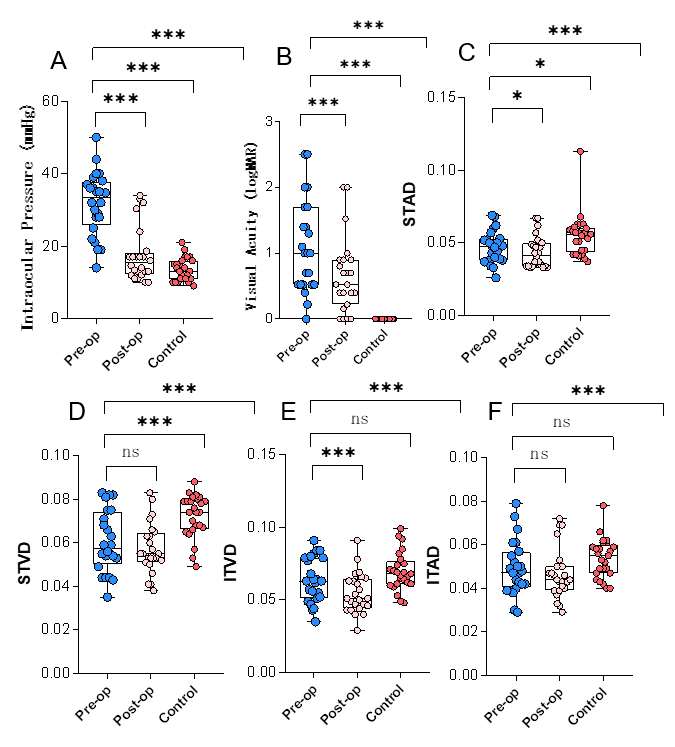

Supplement: Supplementary file 2 [file Image_1.tif]
